# Narrative analysis of interviews conducted with African unaccompanied refugee minors

**DOI:** 10.7189/jogh.15.04174

**Published:** 2025-06-13

**Authors:** Anselm Bründlmayer, Chloe Sales, Ray Zhang, Jennifer Pien, Paul Lukas Plener, Clarissa Laczkovics, Julia Schwarzenberg

**Affiliations:** 1 Department of Child and Adolescent Psychiatry, Medical University of Vienna, Vienna, Austria; 2 University of California San Francisco School of Medicine, San Francisco, California, USA; 3 Columbia University, New York, New York, USA; 4 Department of Psychiatry and Behavioural Sciences, Stanford University School of Medicine, Stanford, California, USA; 5 Department of Child and Adolescent Psychiatry and Psychotherapy, University of Ulm, Ulm, Germany

## Abstract

**Background:**

Unaccompanied refugee minors (URMs) are a particularly vulnerable subgroup of refugees, frequently exhibiting high levels of distress and a wide range of trauma-related symptoms. They are at risk of poor mental health outcomes such as developing complex posttraumatic stress disorder, an emergent mental disorder associated with re-experiencing, hyperarousal, and avoidance of trauma-related stimuli.

**Methods:**

We conducted a qualitative analysis of interviews with 28 unaccompanied refugee minors from African countries residing in Austria.

**Results:**

Three main strands of narrative focus were identified: war-related experiences, present experiences in the new home country, and different coping strategies.

**Conclusions:**

The results of this study suggest that URM narratives are highly complex and that URM require supportive networks and psychotherapeutic care.

Unaccompanied refugee minors (URMs) are children and adolescents aged <18 years who have been separated from both of their parents or caregivers, were involuntarily forced to flee from their respective home countries, and are not being cared for by an adult [1]. In previous studies, URMs have been described as a particularly vulnerable subgroup of refugees with high amounts of suffering and a wide range of symptoms related to posttraumatic stress disorder (PTSD) [2]. An estimated total of about 43 000 URMs sought refuge in Europe in 2023 [3]. Scientific literature to date has given little attention to either quantitative or qualitative research on this culturally diverse population, which exhibits a broad range of symptom manifestations, trauma-related narratives, and psychopathology. This heterogeneous population has thus been described as highly difficult to classify in traditional diagnostic clusters [4]. Due to the frequently precarious and uncertain living circumstances of URMs, it is especially challenging to obtain significant sample sizes in order to conduct traditional epidemiological or intervention studies [5].

URMs are at increased risk of developing psychiatric disorders such as PTSD and depression [6]. PTSD is a common mental disorder following exposure to one or several traumatic events. It is typically characterised by trauma-induced symptoms such as burdensome re-experiencing, increased arousal, and avoidance of potential triggers related to the initial trauma experience [7]. Recent research has identified the following risk factors for poor mental health outcomes in URMs: exposure to trauma, female gender, and older age [8]. In accompanied and unaccompanied refugee minors, symptoms of hyperarousal, nightmares and concentration problems seem to be especially prominent [9].

A review by Reavell et al. found that the prevalence of PTSD varies greatly across studies and ranges from 5–60% [10]. Different levels of resilience and associated factors have been discussed as potential explanatory models for this wide range of prevalence rates. Favourable and supportive living arrangements, as well as sufficient access to mental health institutions and efficient treatment, have been shown to have a positive effect on psychological resilience [11]. Lack of refugee status as well as cumulative traumatic events and daily stressors, have been demonstrated to be significant predictors of PTSD severity [12–14].

There is a frequent co-occurrence of PTSD with other psychiatric disorders, especially major depression, anxiety disorder, substance abuse, personality disorders, and attention deficit hyperactivity disorder [15–17]. Moreover, exposure to trauma has been associated with cognitive deficits and developmental disturbances in minors [18]. Most alarmingly, PTSD is associated with an increased risk of suicidal behaviour. Studies have shown that both adults and minors diagnosed with PTSD exhibit an increased likelihood of attempting or committing suicide [19]. With regards to the newly established International Classification of Diseases 11th revision diagnostic system, the diagnostic entity complex PTSD (cPTSD) has been developed for those who were exposed ‘to an event or series of events of an extremely threatening or horrific nature’ [20], which is often the case in URMs [21]. This new diagnosis emphasises additional symptoms in the realms of affect regulation, negative cognitive bias and social relationships in addition to PTSD symptomatology.

In stark contrast to the aforementioned high symptom load and vulnerability to developing psychiatric disorders among URMs, there is a clear gap in the scientific literature concerning the care needs of this subgroup. This gap points to a lack of suitable, evidence-based interventions for URMs presenting to psychiatric services, and thus, little guidance for clinicians who treat patients of this population. This study is focused solely on African URMs because, at the time of assessment, Austria experienced particularly high numbers of African URMs, indicating a necessity for targeted research and clinical intervention for this subgroup.

Previous research by Thieleman et al. examined trauma-focused cognitive behavioural therapy (TF-CBT) as an intervention for unaccompanied minors to highlight the importance of structured therapeutic frameworks in addressing posttraumatic symptoms [22]. Their work demonstrated TF-CBT’s efficacy, especially for symptoms such as re-experiencing, avoidance, and hyperarousal [22]. While the Thieleman et al. study provided insights into clinical interventions, in this paper, we expand on those findings by shifting the focus from symptom-based treatment to the rich, multifaceted narratives shared by these minors.

Additionally, this study builds upon the previous paper by Huemer et al. by expanding the scope of analysis to capture a broader and more complex range of experiences among the URMs [23]. Huemer et al. analysed psycholinguistic variables to assess emotional expressiveness and avoidance in URM narratives [23], whereas we adopted a qualitative approach to explore broader narrative themes, including war-related experiences, present life in a new country, and coping mechanisms. This focus provides a more comprehensive view of both the challenges and resilience factors in these minors’ lives. By emphasising the lived experiences of African URMs, this study aims to inform therapeutic and culturally sensitive interventions, offering a more nuanced understanding of this vulnerable population.

The aim of this study was to disclose narrative focuses of traumatised unaccompanied refugee minors in order to further identify the specific needs of this particular subgroup. The following subordinate research objectives fulfilled the purpose of reference points to guide the research process: 1) establishing a common framework that connects diverse strands of trauma-related narratives, 2) identifying differences and similarities in a heterogeneous set of narratives, and 3) describing areas of potential psychosocial support and interventions.

## METHODS

The data used in this study were previously examined by Huemer et al., focusing on specific psycholinguistic variables linked to emotional expressivity and narrative style [23]. Pointing to an insufficient integration of trauma among URMs, it showed higher referential activity (level of transmission of non-verbal experiences in spoken language) and fewer temporal junctures (two past-tense clauses that correspond to the chronological order of the narrated events) in the free association task compared to an age-matched control group [23]. For the purposes of this study, we attempted to establish a study design focusing on qualitative content analysis.

We obtained 28 interview transcripts from a larger project on mental health issues among African unaccompanied refugee minors residing in Austria. With the help of a non-governmental organisation that provided counselling for migrants and refugees, all residential accommodations (housing centres) under the responsibility of the Austrian public welfare system, which were accommodating asylum seekers financed by the Austrian state, were invited to participate in the study. The study and its instruments were presented to the people in charge of each participating institution, and their administrative consent was obtained. Eight out of 15 eligible residential sites were included. Further, 41 participants fulfilled the inclusion criteria (being a URM from an African country). Of these, 35 were male and six were female. A total of 28 subjects agreed to participate. Countries of origin that were represented in the study were the Gambia, Somalia, Nigeria, Kenya, Ghana, and Eritrea [23–26]. Further details on participant recruitment, including selection criteria for the housing centres and any differences between sites, are provided in an earlier publication [25].

The 28 participants agreed to undergo the stress-inducing speech task, a structured interview style that is widely recognised in literature as a validated, standardised protocol for eliciting trauma narratives [27]. In this standardised protocol, which has been successfully and safely implemented in previous studies, the participants were asked to complete two balanced tasks within a 10-minute time frame: speaking about anything that comes to their mind (free association condition (FA)) and their most stressful life event (stress condition (STR)) [27]. Randomisation determined whether each participant started with the STR condition or the FA condition. After instructions were given, the subjects performed the two speech tasks without interruption. A researcher who recorded the interviews was the only other person present [25]. Under direct clinical supervision, the team was prepared to address any acute stress responses among the adolescents by using standardised debriefing measures. All interviews were transcribed verbatim by the principal investigator of the original study. While 25 interviews were conducted in English, three transcripts were translated into English from German or French, respectively.

The speech task was originally designed to produce a mild degree of stress for the purposes of measuring stress response in children and adolescents, and this controlled stress often prompted participants to share more emotional details, allowing for richer, more authentic narratives [27]. For this study, the speech task was combined with the FA task to obtain sufficient access to URM narratives.

This paper draws on data obtained initially for a larger epidemiological study on mental health among URMs in Austria. After a complete description of the study was given to the subjects, written informed consent was obtained. The questionnaires were distributed by trained personnel, who also offered instructions and guidance throughout the assessment. The committee of the Medical University of Vienna approved this study (approval number 246/2005).

### Qualitative approach

There are several challenges in studying vulnerable youth subgroups like URM; larger sample sizes to enable quantitative data are thus hard to obtain. Qualitative methods enable the empirical study of these high-risk populations while also obtaining data in a less formalised and more grounded way [27]. By utilising qualitative methods, multi-layered information that covers topics such as culture, beliefs, attitudes, and individual experiences can be gathered and utilised to complement psycho-epidemiological research. For example, a mixed-method study by Hanewald et al. analysed the current mental condition in UMR by integrating qualitative data from narrative biographical interviews of UMR with psychometric screening data, identifying differentiating factors influencing the mental health issues within the URM cohort [28]. Thus, extending knowledge and understanding of multi-faceted URM narratives may increase the efficacy of planned interventions.

In this study, using a grounded theory qualitative approach, we established a code structure with initial open codes and subsequent axial codes. We applied guidelines obtained from the SAGE Handbook of Qualitative Research to ensure a scientifically valid procedure [29]. The purpose of the model, composed of various codes, was to elucidate narrative focuses detected in the anonymised interview transcripts.

Authors AB and JS established the coding structure by means of consensus coding. Each transcript was reviewed on a one-to-one basis in a joint effort. During the initial coding phase, we identified passages in the transcript that contained narrative strands. After describing the content of these marked sections, we recognised common themes featured in multiple transcripts and we used open and axial coding and discussed them in consensus meetings. Theoretical saturation was achieved by reaching sufficient overlap and repetition of narrative themes corresponding to the initial draft of the code tree. The codebook was then reviewed and discussed with author CL, who had previously not been involved in data analysis, in order to provide external validation and reduce bias. In this step, the subcodes ‘Facing racism’ and ‘Current living situation’ were subsumed into the subcode ‘Experiences with others’ since significant overlap between those categories and a shared emphasis on interpersonal relations were identified. This was discussed in further consensus meetings.

We used software NVivo, version 12.3.0 (Lumivero, Denver, Colorado, USA) to establish a coding system by gradually identifying important themes that were translated into codes and corresponding axial subcodes.

## RESULTS

We detected three distinct groups of narrative focuses. The first main narrative strand addressed experiences related to war. Subjects spoke about their experiences of living under such circumstances, how it related to their flight, and the severe traumatic effects of war-related events.

**Figure 1:**
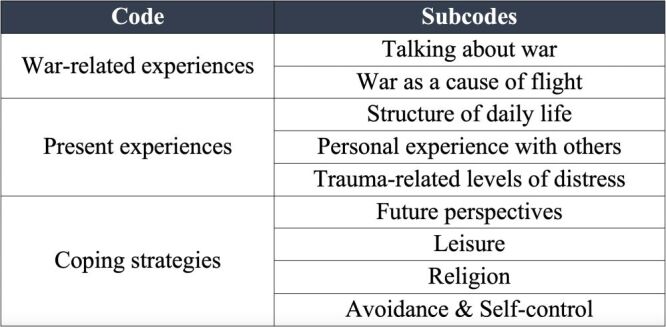
Coding tree with narrative focuses.

The second narrative focus drew upon the subjects' present experience of life in a new country. It included descriptions of their drastically changed living situation, the problems they face, and how trauma-induced distress is still present even after seeking refuge. The third narrative strand explored the coping strategies and resilience factors established by the subjects in order to process traumatising events and persistent present distress (**Figure 1**).

### War-related experiences

The first main narrative strands comprised experiences related to war. Some URM participants spoke in detail about their observations. They reported on experiences regarding violence, death threats, blackmailing, death of family members, and shootings. They described high levels of dismay and constant fear of death.

While this share of participants depicted war and its strains in great depth, others relied on more superficial descriptions and refrained from sharing stress-inducing details. In those cases, war was depicted as an abstract concept. Without elaborating on concrete details, they still emphasised the highly burdensome qualities of war-related events. URM subjects frequently perceived exposure to war as highly stressful, leading to a high symptom load related to mental health. War exposure was described as highly threatening to their integrity and exceeded their cognitive processing capacities. They reported flashbacks to occur suddenly and were often triggered by increased levels of stress (**Table 1**).

**Table 1:** Extract from subcode ‘Talking about war’

Items
**Code**
War-related experiences
**Subcode**
Talking about war
**Quote**
*‘They don’t want to say to my custodian that my mother is dead. They were hiding it from me. But later I found out that she is dead. That is it and also here, the time I came here, I used to cry all the time, feeling sad so much and sometimes when I cool down then sometimes it comes again. Sometimes it stops but when someone makes trouble with me, then I used to remind all that things that happened before (...)’*

Many URM participants described war as the prime reason for fleeing from their home country, which forced them to overcome risky escape routes and resettle in a new country. In those cases, war-related experiences were so devastating and severe that they were left with no other choice than to leave their country unaccompanied by adult caregivers.

Participants identified multiple war-related factors that contributed to stress levels in their past lives. Daily errands such as using transit or obtaining food became nearly impossible tasks. War also prevented the subjects from pursuing routine developmental tasks such as recreation, personal relationships, friendships, and regular school attendance. Furthermore, a constant underlying fear of the possibility of experiencing violence was described. Living in a war zone was often experienced as a latent threat in which the fear of death was omnipresent.

### Present experiences

Many URM participants were open about sharing their current life experiences, including personal stories in their new surroundings and interactions with others. These reports focused on adapting to new living situations and continuing to face challenges in an unfamiliar society, despite considerable time spent in Austria. These problems were often mentioned when discussing daily life, for example, when running errands.

While trying to adapt to a foreign culture posed a difficult challenge for many URM participants, they also described living in Austria as a significant relief. Having endured living in war-torn countries, they often felt as though they had finally arrived in a safe space. Furthermore, they mentioned that the welfare state addressed their needs and ensured their personal well-being. However, URM subjects often found themselves as targets of racism. Some subjects reported the occurrence of racial profiling, for example, being suspected of criminal activities by the authorities based on their appearance and/or skin colour. Besides concrete experiences of racism in daily life, URMs also described more subtle forms of racism. There was a frequent perception of locals’ reluctance to interact with the subjects due to their skin colour. They frequently described this feeling as a main factor not only for the lack of social acceptance and inclusion, but also for explicit social exclusion (**Table 2**).

**Table 2:** Extract from subcode ‘Facing racism’

Items
**Code**
Present experiences
**Subcode**
Personal experiences with others
**Quote**
*‘Here there is no equal chance for the blacks and that really sucks and, and racism is not very good here because to me it’s something that always worries me. No friends and especially living in a country which is not an English-speaking country. Many people speak English here but they are always expecting you to speak their language. Even when you can talk to them in their language, someone just looks at you and starts to run away. Someone even looks at you standing far away from you and would just call the police, say there’s a black man here which is all not good.’*

According to several subjects, the severe lack of structure in their daily lives was a major barrier to their well-being. Many were unable to attend school regularly or undergo job training. Due to a lack of financial means, they also found it difficult to engage in social activities, such as joining leisure or sports clubs. As a result, subjects experienced boredom and disorientation. Some reported that it was common for them to merely sit around in their homes because they lacked access to meaningful activities. Particularly regarding school, many of the URMs were well aware of the importance of education. The desire for regular school attendance was especially persistent, considering that many of the subjects could not enjoy regular school attendance in their countries of origin.

Many subjects also reported high levels of distress that accumulated in response to various burdensome triggers. In particular, the subjects who were directly affected by war-related violence, or who had to leave their country due to political or religious conflicts, appeared to be under considerable stress. Often, they associated these high levels of distress with losing their family members, which subsequently resulted in a lack of psychological attachment figures. Many subjects shared that the lack of information about the whereabouts of certain family members caused high degrees of uncertainty regarding their well-being. Others were certain that some of their close relatives had been murdered. In both cases, solitude resulting from absent caregivers was another factor that caused high levels of distress. Some of the URMs participants were also able to identify certain sensitive trigger points related to sudden changes in stress levels, putting them at increased risk of externalising symptoms such as aggressive behaviour.

### Coping strategies

The third narrative focus included the wide range of coping strategies and resilience factors to which URMs resort. Some coping strategies were more deliberate (*e.g.* sports) while others stemmed from underlying compensation mechanisms (*e.g.* avoidant behaviour). Many of the URMs put great emphasis on establishing meaningful future perspectives. Reflecting on the future helped them restructure their lives, which were often dominated by challenging socio-economic circumstances. Several URM participants were hopeful that establishing a future framework would help them manage their burdensome past and their difficulties in adapting to a new society.

Focusing on meaningful perspectives had implications for different areas of the lives of URMs. Many subjects wanted to learn the language of their new home country or improve their already existing language skills. Learning German was seen as particularly important for adapting to a society that alienated them and improving their professional careers. Several participants were hopeful about being able to attend school regularly. To some participants, this was a particularly crucial aspect as they were unable to regularly acquire knowledge in educational institutions while living in their home countries.

Another important future perspective was finding work. Several study participants related being employed to the ability to stay in Austria on a long-term basis. Besides language proficiency, education, and work, some URM participants identified future goals that were associated with psychological growth. They wanted to overcome their burdensome past by successfully processing their traumatic experiences and being able to talk about those events (**Table 3**). A large portion of the study participants mentioned that leisure plays an important role in coping with their stressful past and debilitating intrusive thoughts. Spending time with other people was reported to be particularly helpful. A supportive social network helped participants overcome the burdens of daily life, especially when interacting with people who had experienced similar war-related life events.

**Table 3:** Extract from subcode ‘Future perspectives’

Items
**Code**
Coping strategies
**Subcode**
Future perspectives
**Quote**
*‘The law says it is not written in my papers to work. So this is why I lost this job and now I need a job if I want to stay in this country. I want for me to pay taxes in Austria, think that it can be possible so please I need help. I need, I want to make work and before I work I need to make school and so that I understand the language that is more important. If I can’t speak the language for me to stay in the country it will be difficult. I must speak their language (...)’*

Sport (especially football) was also frequently used as a method of distraction when emotional strains became too overwhelming. Other leisure activities, such as watching television or listening to music, fulfilled a mainly distractive function. On multiple occasions, religion and spirituality were mentioned as coping strategies that were well-incorporated into the lives of the study participants. Coping strategies discussed included how their upbringing was profoundly intertwined with a constant presence of faith. To them, their belief in God had always been an important element in their lives and thus still fulfilled an identity-establishing and supportive function.

Some URM subjects discussed how their faith and spirituality served as an important framework through which they attempted to describe and reconstruct their past. Religious ideas were used to establish narratives and explanatory models dealing with the manner in which their biographies had proceeded. In the context of religion, participants were often thankful that they were still alive and had managed to survive living in war-torn countries. A considerable number of participants relied on defense mechanisms related to avoidant and self-controlling behaviour. Some had the tendency to withdraw from social life and preferred to stay at home while refraining from social activities. However, this strategy frequently proved unsuccessful at combating PTSD symptoms such as intrusive thoughts and flashbacks. The ability to avoid thoughts about traumatic events was often associated with high degrees of self-control. These URMs tried to contain and manage their past by rationalising their experiences of violence without emotionally accessing them.

Some URM subjects exhibited highly avoidant behaviour when asked to disclose burdensome events from their pasts. One participant refused to take part in the stress condition of the speech task, and several gave short answers or made general statements without sharing any details. Many URMs emphasised that there was a significant lack of possibilities to discuss their traumatic experiences. There was no dedicated framework for them to process thoughts related to trauma. Consequently, instead of being left alone with those overwhelming thoughts, they preferred to avoid them in the first place.

## DISCUSSION

In this study, we presented an in-depth analysis of narratives by African unaccompanied refugee minors. To improve our understanding, a grounded theory qualitative method approach was chosen. The main objective was to disclose the narratives and specific needs of a complex and diverse population. While symptom-based frameworks such as the ICD-11 have incorporated cPTSD with an emphasis on child soldiers and genocide survivors, significant gaps still remain in translating diagnostic categories into care for refugee youth [30]. Thus, the focus of this study was shifted to underlying and more subtle elements related to trauma and PTSD that are frequently overlooked in self-report scales and trauma questionnaires. This research is particularly essential, as recent evidence from a study using a network approach showed a variety of re-experiencing symptoms in URM populations [9,31].

For clinicians and other health care professionals, developing nuanced listening skills is crucial when working with a vulnerable subgroup that lacks public advocacy, such as unaccompanied refugee minors. Identifying meaningful cues within the scope of trauma narration may facilitate the development of appropriate diagnostic procedures as well as subsequent treatment strategies. This could be crucial in further tailoring specific approaches using narratives, such as trauma-focused therapy, to the needs of URM populations [23].

The results indicated that the perception of war and the witnessing of brutal violence played a crucial role in URM narratives. There was a great variety in the way war-related events were reported. Some participants depicted war in a largely superficial manner, while others shared their experiences in detail. Most URM participants mentioned that the experience of war-related events was perceived as highly stressful and threatening. Acts of violence and the deaths of family members were described as severely incisive. They were also associated with high levels of PTSD-related symptoms such as intrusive thoughts and hyperarousal, symptoms commonly encountered in this population [9]. These findings are relevant on a global scale, given ongoing trends in forced migration. According to recent United Nations High Commissioner for Refugees data, nearly two-thirds of all refugees originate from just four countries, and almost 50% of those displaced are children aged <18 years [32]. In this context, psychotherapy serves a vital function by providing a framework that functions as a safe space for the disclosure of traumatising events.

In previous studies, the efficacy of psychotherapy in trauma-related disorders has been well-established [33]. Unterhitzenberger et al. have suggested that trauma-focused cognitive behavioural therapy is a highly efficient way of treating URMs with PTSD [34]. Pasupathi et al. have shown that active narration of trauma-related experiences is a useful tool to reduce distress among youth [35]. They suggested that the underlying mechanisms behind narration-induced stress relief stem from the ability to draw meaning from negative events. A structured setting and professional guidance are, therefore, crucial to managing such cognitive processes. Another study has suggested that narration is effective at down-regulating negative emotions [36].

While there is strong evidence regarding the efficacy of cognitive behavioural therapy, establishing access to psychotherapy in low-resource settings has proven challenging for URMs [34]. Residential institutions could encourage access to cognitive behavioural therapy to help URM overcome administrative and financial obstacles, and group interventions in youth residential care seem to hold potential for young refugees. Furthermore, digital interventions might show some promise in providing scalable treatments. However, to date, there is still limited evidence for their efficacy [37]. Legislative directives must address these needs, for instance, by determining mentoring ratios or enabling access to work permits and education [38]. The participants also addressed various modes of coping strategies and mechanisms that supported cognitive processing of trauma-related events. One of the main coping strategies mentioned was the establishment of structures and future perspectives. Focusing on potential future perspectives may, therefore, indeed reduce negative affect among URMs.

These findings point to the great importance of the living conditions in which URMs find themselves. Previous studies have suggested that supportive living conditions effectively reduce posttraumatic symptoms. Ideally, they should be highly structured environments supervised by a multi-professional team offering an immediate network of psycho-social support. It has also been suggested that sufficient freedom of movement and the absence of rigorous restriction reduce anxiety-related symptoms [10].

Furthermore, residential institutions should focus on providing access to language learning activities. Several study participants identified the acquisition of the local language as a crucial aspect of social inclusion. At the same time, meaningful networks in which URMs maintain connections to the cultures of their countries of origin should be encouraged. In particular, religion and spirituality were important factors in coping with stressful memories among study participants. Such coping strategies should be regarded as an asset that strengthens resilience and should not be taken away at the expense of unilateral adaptation to the new home country.

URMs should also receive support in finding employment or job training, which was identified as a key factor in establishing a structured future perspective and which served as a highly efficient coping strategy. Interestingly, it has been reported that the majority of a nationally representative German population sample agree that URM who have finished school or an apprenticeship in their host country should be granted permission to stay permanently [39]. This finding supports an alignment between the need for education expressed by URMs and the expectations expressed by their host country’s population.

While some URMs exhibited healthy coping mechanisms such as leisure, other study participants reported on negative coping styles such as highly avoidant behaviour. Previous studies have suggested that distinct modes of coping styles and/or defence mechanisms are associated with different levels of psychological resilience [40]. Additionally, family support, including maintaining consistent routines and providing emotional reassurance, was identified as an effective method of decreasing emotional distress [33]. As suggested in our findings, there is a great emphasis on coping strategies that draw upon external structures and stability. This is particularly important as severe trauma is frequently associated with disintegration and fragility of inner structures [41]. Thus, external factors that provide structure may play a vital role in stabilising the inner self and promoting resilience among URMs. An attempt to improve mental health outcomes would therefore be to not only offer psychotherapeutic interventions but also sufficient psychosocial support and availability of resources that provide stability and security [38].

### Strengths and limitations

One major limitation of the study is the relatively small sample size (n = 28). Due to the vulnerable social and economic situation of URMs, they are a particularly difficult subgroup to recruit for study purposes. As a result, the data obtained may not sufficiently depict the current composition of the study population of interest. Moreover, focusing on African URM may also limit the generalisability of the findings to other refugee groups, and translating three interviews from German or French into English could introduce subtle shifts in meaning that affect the interpretation of certain narratives. This is particularly important considering annual URM figures have significantly increased compared to the time of data collection [1].

While causal assumptions could not be derived from the results, the study attempted to demonstrate a deeper understanding of URMs as a vulnerable subgroup. Future interventional studies or randomised control trials involving URMs should thus be carefully planned. The advantage of a qualitative approach may contribute to a more nuanced understanding of URMs that extends beyond traditional diagnostic frameworks.

Future studies should also focus on collecting new data to highlight changes within the subgroup of URMs. Addressing residential homes dedicated to housing URMs has proven challenging for administrative reasons. In a study focusing on URMs in Sweden, Ramel et al. found that URMs are significantly overrepresented in psychiatric inpatient care [42]. It might therefore be advisable for future studies to focus on recruiting participants within the scope of clinical settings.

## CONCLUSIONS

In summary, we examined URM narratives using a grounded theory qualitative approach. The three main narrative focuses included war-related experiences, present life, and coping strategies of URMs. Trauma-related issues described in this study could be tackled by providing supportive living circumstances, future perspectives (access to work and education) and facilitated access to psychotherapy, which can establish a protected framework for narration and reduce posttraumatic symptoms. Future interventions may benefit from qualitative findings related to URM narratives.

## References

[1] United Nations High Commissioner for Refugees. Mid-Year Trends 2016. 2016. Available: http://www.refworld.org/reference/annualreport/unhcr/2016/en/115883. Accessed: 29 October 2024.

[2] Witt A Rassenhofer M Fegert JM Plener PL [Demand for help and provision of services in the care of unaccompanied refugee minors: A systematic review]. Kindheit und Entwicklung. Zeitschrift für Klinische Kinderpsychologie. 2015;24:209–24. German. 10.1026/0942-5403/a000177

[3] European Union Agency for Asylum. Data Analysis of Unaccompanied Minors in 2023, Fact Sheet No. 29. Grand Harbour, Malta: European Union Agency for Asylum; 2024. Available: http://euaa.europa.eu/sites/default/files/publications/2024-08/2024_factsheet29_data_unaccompanied_minors_EN.pdf. Accessed: 29 October 2024.

[4] Rousseau C Said TM Gagné MJ Bibeau G Resilience in unaccompanied minors from the north of Somalia. Psychoanal Rev. 1998;85:615–37. 9870245

[5] Pieloch KA McCullough MB Marks AK Resilience of children with refugee statuses: A research review. Canadian Psychology / Psychologie Canadienne. 2016;57:330–9. 10.1037/cap0000073

[6] Smid GE Lensvelt-Mulders GJLM Knipscheer JW Gersons BPR Kleber RJ Late-Onset PTSD in Unaccompanied Refugee Minors: Exploring the Predictive Utility of Depression and Anxiety Symptoms. J Clin Child Adolesc Psychol. 2011;40:742–55. 10.1080/15374416.2011.597083 21916692

[7] Friedman MJ, Keane TM, Resick PA. Handbook of PTSD: Science and practice: 2nd ed. New York, New York, USA: The Guilford Press; 2014.

[8] Bamford J Fletcher M Leavey G Mental Health Outcomes of Unaccompanied Refugee Minors: a Rapid Review of Recent Research. Curr Psychiatry Rep. 2021;23:46. 10.1007/s11920-021-01262-8 34196826 PMC8249279

[9] Pfeiffer E Sukale T Müller LRF Plener PL Rosner R Fegert JM The symptom representation of posttraumatic stress disorder in a sample of unaccompanied and accompanied refugee minors in Germany: A network analysis. Eur J Psychotraumatol. 2019;10:1675990. 10.1080/20008198.2019.1675990 31681465 PMC6807914

[10] Reavell J Fazil Q The epidemiology of PTSD and depression in refugee minors who have resettled in developed countries. J Ment Health. 2017;26:74–83. 10.1080/09638237.2016.1222065 27684305

[11] Mitra R Hodes M Prevention of psychological distress and promotion of resilience amongst unaccompanied refugee minors in resettlement countries. Child Care Health Dev. 2019;45:198–215. 10.1111/cch.12640 30661259

[12] Knipscheer JW Sleijpen M Mooren T Ter Heide FJJ van der AN. Trauma exposure and refugee status as predictors of mental health outcomes in treatment-seeking refugees. BJPsych Bull. 2015;39:178–82. 10.1192/pb.bp.114.047951 26755950 PMC4706143

[13] Vervliet M Lammertyn J Broekaert E Derluyn I Longitudinal follow-up of the mental health of unaccompanied refugee minors. Eur Child Adolesc Psychiatry. 2014;23:337–46. 10.1007/s00787-013-0463-1 23979476

[14] Hornfeck F Eglinsky J Garbade M Rosner R Kindler H Pfeiffer E Mental health problems in unaccompanied young refugees and the impact of post-flight factors on PTSS, depression and anxiety - A secondary analysis of the Better Care study. Front Psychol. 2023;14:1149634. 10.3389/fpsyg.2023.1149634 37408964 PMC10318408

[15] Spinhoven P Penninx BW van Hemert AM de Rooij M Elzinga BM Comorbidity of PTSD in anxiety and depressive disorders: Prevalence and shared risk factors. Child Abuse Negl. 2014;38:1320–30. 10.1016/j.chiabu.2014.01.017 24629482

[16] Biederman J Petty CR Spencer TJ Woodworth KY Bhide P Zhu J Examining the nature of the comorbidity between pediatric attention deficit/hyperactivity disorder and post-traumatic stress disorder. Acta Psychiatr Scand. 2013;128:78–87. 10.1111/acps.12011 22985097 PMC3527641

[17] Simmons S Suárez L Substance Abuse and Trauma. Child Adolesc Psychiatr Clin N Am. 2016;25:723–34. 10.1016/j.chc.2016.05.006 27613348

[18] Malarbi S Abu-Rayya HM Muscara D Stargatt R Neuropsychological functioning of childhood trauma and post-traumatic stress disorder: A meta-analysis. Neurosci Biobehav Rev. 2017;72:68–86. 10.1016/j.neubiorev.2016.11.004 27851897

[19] Dobry Y Sher L The underexamined association between posttraumatic stress disorder, medical illness and suicidal behavior. Int J Adolesc Med Health. 2013;25:279–82. 10.1515/ijamh-2013-0063 24006323

[20] World Health Organization. ICD-11 for Mortality and Morbidity Statistics. 2024. Available: http://icd.who.int/browse/2024-01/mms/en#585833559. Accessed: 29 October 2024.

[21] Lotzin A Morozova-Larina O Paschenko S Paetow A Schratz L Keller V War-related stressors and ICD-11 (complex) post-traumatic stress disorders in Ukrainian students living in Kyiv during the Russian-Ukrainian war. Psychiatry Res. 2023;330:115561. 10.1016/j.psychres.2023.115561 37956590

[22] Thielemann JFB Kasparik B König J Unterhitzenberger J Rosner R A systematic review and meta-analysis of trauma-focused cognitive behavioral therapy for children and adolescents. Child Abuse Negl. 2022;134:105899. 10.1016/j.chiabu.2022.105899 36155943

[23] Huemer J Nelson K Karnik N Völkl-Kernstock S Seidel S Ebner N Emotional expressiveness and avoidance in narratives of unaccompanied refugee minors. Eur J Psychotraumatol. 2016;7:29163. 10.3402/ejpt.v7.29163 26955827 PMC4783431

[24] Huemer J Karnik N Völkl-Kernstock S Granditsch E Plattner B Friedrich M Psychopathology in African Unaccompanied Refugee Minors in Austria. Child Psychiatry Hum Dev. 2011;42:307–19. 10.1007/s10578-011-0219-4 21293919

[25] Huemer J Völkl-Kernstock S Karnik N Denny KG Granditsch E Mitterer M Personality and Psychopathology in African Unaccompanied Refugee Minors: Repression, Resilience and Vulnerability. Child Psychiatry Hum Dev. 2013;44:39–50. 10.1007/s10578-012-0308-z 22661148

[26] Huemer J Karnik N Völkl-Kernstock S Granditsch E Dervic K Friedrich MH Mental health issues in unaccompanied refugee minors. Child Adolesc Psychiatry Ment Health. 2009;3:13. 10.1186/1753-2000-3-13 19341468 PMC2682790

[27] Steiner H Ryst E Berkowitz J Gschwendt MA Koopman C Boys’ and girls’ responses to stress: affect and heart rate during a speech task. J Adolesc Health. 2002;30:14–21. 10.1016/S1054-139X(01)00387-1 11943570

[28] Hanewald B Knipper M Fleck W Pons-Kühnemann J Hahn E Ta TMT Different Patterns of Mental Health Problems in Unaccompanied Refugee Minors (URM): A Sequential Mixed Method Study. Front Psychiatry. 2020;11:324. 10.3389/fpsyt.2020.00324 32411027 PMC7198874

[29] Denzin NK, Lincoln YS. The SAGE Handbook of Qualitative Research. Thousand Oaks, California, USA: SAGE; 2005.

[30] Brewin CR Complex post-traumatic stress disorder: a new diagnosis in ICD-11. BJPsych Adv. 2020;26:145–52. 10.1192/bja.2019.48

[31] Hashemi B Shaw RJ Hong DS Hall R Nelson K Steiner H Posttraumatic stress disorder following traumatic injury: Narratives as unconscious indicators of psychopathology. Bull Menninger Clin. 2008;72:179–90. 10.1521/bumc.2008.72.3.179 18990054

[32] United Nations High Commissioner for Refugees. Refugee Data Finder. 2024. Available: http://www.unhcr.org/refugee-statistics. Accessed: 29 October 2024.

[33] Kerbage H Bazzi O El Hage W Corruble E Purper-Ouakil D Early interventions to prevent post-traumatic stress disorder in youth after exposure to a potentially traumatic event: A scoping review. Healthcare (Basel). 2022;10:818. 10.3390/healthcare10050818 35627955 PMC9141228

[34] Unterhitzenberger J Eberle-Sejari R Rassenhofer M Sukale T Rosner R Goldbeck L Trauma-focused cognitive behavioral therapy with unaccompanied refugee minors: a case series. BMC Psychiatry. 2015;15:260. 10.1186/s12888-015-0645-0 26497391 PMC4619299

[35] Pasupathi M Wainryb C Mansfield CD Bourne S The feeling of the story: Narrating to regulate anger and sadness. Cogn Emot. 2017;31:444–61. 10.1080/02699931.2015.1127214 26745208 PMC5584785

[36] Wainryb C Pasupathi M Bourne S Oldroyd K Stories for All Ages: Narrating Anger Reduces Distress Across Childhood and Adolescence. Dev Psychol. 2018;54:1072–85. 10.1037/dev0000495 29553770 PMC5962367

[37] Schepp S Fegert JM Rassenhofer M Regner S Witt A Pfeiffer E Evaluation of ANKOMMEN as a group intervention based on life story work for adolescents in residential care in Germany: A single-arm pilot study. Child Adolesc Psychiatry Ment Health. 2024;18:135. 10.1186/s13034-024-00817-w 39438937 PMC11515701

[38] Silove D Ventevogel P Rees S The contemporary refugee crisis: an overview of mental health challenges. World Psychiatry. 2017;16:130–9. 10.1002/wps.20438 28498581 PMC5428192

[39] Plener PL Groschwitz RC Brähler E Sukale T Fegert JM Unaccompanied refugee minors in Germany: attitudes of the general population towards a vulnerable group. Eur Child Adolesc Psychiatry. 2017;26:733–42. 10.1007/s00787-017-0943-9 28074291 PMC5446565

[40] Hooberman J Rosenfeld B Rasmussen A Keller A Resilience in trauma-exposed refugees: the moderating effect of coping style on resilience variables. Am J Orthopsychiatry. 2010;80:557–63. 10.1111/j.1939-0025.2010.01060.x 20950296

[41] Farina B Imperatori C Are traumatic disintegration, detachment, and dissociation separate pathogenic processes related to attachment trauma? A working hypothesis for clinicians and researchers. Psychopathology. 2024;57:236–47. 10.1159/000535191 38071976

[42] Ramel B Täljemark J Lindgren A Johansson BA Overrepresentation of unaccompanied refugee minors in inpatient psychiatric care. Springerplus. 2015;4:131. 10.1186/s40064-015-0902-1 25825687 PMC4372620

